# Deletion of RAGE fails to prevent hepatosteatosis in obese mice due to impairment of other AGEs receptors and detoxifying systems

**DOI:** 10.1038/s41598-021-96859-7

**Published:** 2021-08-30

**Authors:** Kristiaan Wouters, Alessia S. Cento, Katrien H. Gaens, Margee Teunissen, Jean L. J. M. Scheijen, Federica Barutta, Fausto Chiazza, Debora Collotta, Manuela Aragno, Gabriella Gruden, Massimo Collino, Casper G. Schalkwijk, Raffaella Mastrocola

**Affiliations:** 1 Department of Internal Medicine, MUMC, Maastricht, Limburg The Netherlands; 2 Cardiovascular Research Institute Maastricht, Maastricht, Limburg The Netherlands; 3 Department of Clinical and Biological Sciences, University of Turin, Corso Raffaello 30, 10125 Turin, Italy; 4 Department of Medical Sciences, University of Turin, Turin, Italy; 5 Department of Drug Sciences, University of Eastern Piedmont, Novara, Italy; 6 Department of Drug Science and Technology, University of Turin, Turin, Italy

**Keywords:** Non-alcoholic fatty liver disease, Obesity, Dyslipidaemias, Fat metabolism, Mechanisms of disease, Inflammation, Inflammasome

## Abstract

Advanced glycation endproducts (AGEs) are involved in several diseases, including NAFLD and NASH. RAGE is the main receptor mediating the pro-inflammatory signalling induced by AGEs. Therefore, targeting of RAGE has been proposed for prevention of chronic inflammatory diseases. However, the role of RAGE in the development of NAFLD and NASH remains poorly understood. We thus aimed to analyse the effect of obesity on AGEs accumulation, AGE-receptors and AGE-detoxification, and whether the absence of RAGE might improve hepatosteatosis and inflammation, by comparing the liver of lean control, obese (LeptrDb−/−) and obese RAGE-deficient (RAGE−/− LeptrDb−/−) mice. Obesity induced AGEs accumulation and RAGE expression with hepatosteatosis and inflammation in LeptrDb−/−, compared to lean controls. Despite the genetic deletion of RAGE in the LeptrDb−/− mice, high levels of intrahepatic AGEs were maintained accompanied by decreased expression of the protective AGE-receptor-1, impaired AGE-detoxifying system glyoxalase-1, and increased expression of the alternative AGE-receptor galectin-3. We also found sustained hepatosteatosis and inflammation as determined by persistent activation of the lipogenic SREBP1c and proinflammatory NLRP3 signalling pathways. Thus, RAGE targeting is not effective in the prevention of NAFLD in conditions of obesity, likely due to the direct liver specific crosstalk of RAGE with other AGE-receptors and AGE-detoxifying systems.

## Introduction

Advanced glycation endproducts (AGEs) are toxic compounds produced from reactions between proteins and sugars or fatty acids^1^. AGEs are known to be involved in the development of diabetic complications through receptor-mediated activation of intracellular inflammatory signalling^2^. Recently, accumulation of AGEs in adipose tissue has been linked to the onset of metabolic diseases^3,4^. In particular, plasma carboxymethyllysine (CML), one of the best characterized AGEs, has been demonstrated to be preferentially trapped in adipose tissue through the binding with the receptor for AGEs (RAGE)^5^, leading to the activation of proinflammatory and prooxidant signalling that can interfere with peripheral insulin sensitivity^6^.

RAGE is a member of the superfamily of Ig molecules and belongs to the class of type I cell-surface receptors^7^. RAGE engages numerous ligands including AGEs and exhibits broad expression on many cell types^8^. Generally, RAGE activates NFkB, but its expression is in turn under control of NFkB, thus representing a positive feedback for further increased RAGE expression^9,10^. In a physiological state RAGE is expressed at low levels^11^, but pathophysiological conditions, such as diabetes, chronic inflammation, or obesity, induce increased RAGE expression in different tissues, such as vasculature, adipose tissue and liver^10^. Although sustained activation of RAGE during pathological events results in inflammation and promotes the development of complications, targeting RAGE as a therapeutic treatment remains controversial because in physiological conditions RAGE plays a key role in both innate and adaptive immune responses^12^.

Genetic deletion of RAGE has been widely used to investigate the beneficial effects of RAGE targeting. The deletion of RAGE in rodent models of diabetes and diet-induced obesity has been demonstrated to be effective in reduction of vascular complications^13^, atherosclerosis^14,15^, kidney disease^16,17^, and synaptic injury in brain^18^. Moreover, in the obese mouse model LeptrDb−/−, the genetic deletion of RAGE has been shown to reduce CML accumulation in adipose tissue, as well as circulating cytokines and adipokines, ameliorating insulin resistance^5^.

However, little is known about the effect of RAGE deletion in liver, which is one of the main organs involved in glucose and lipid metabolism. Indeed, the expression of RAGE is often reported to be increased in association to AGEs accumulation during the development of hepatic steatosis and inflammation in both animals and humans, suggesting a key role for the AGE-RAGE signalling in NAFLD and NASH^19,20^. Specifically, it has been proposed that AGEs could contribute to the progression from NAFLD to NASH by RAGE-dependently activating pro-inflammatory and pro-fibrotic signallings. However, since AGEs levels are already elevated upon the appearance of steatosis, we may speculate that AGEs exert a primary role in the onset of NAFLD by affecting hepatic lipid metabolism^19,21^.

However, conflicting data have been reported about the prominent role of RAGE in the development of these diseases and the efficacy of RAGE deletion, depending on the experimental model used^22^. It has been demonstrated that the blockade of RAGE in CML-treated hepatocytes prevents hepatocytes inflammation^20^ and that hepatocyte-specific RAGE deletion attenuates NASH in an experimental mouse model fed with a high AGEs diet^23^. On the contrary, whole-body deletion of RAGE in a model of cholesterol-induced NASH in hyperlipidemic mice^24^ did not improve either lipids accumulation or inflammation^22^, indicating that AGEs may participate in the development of NAFLD and NASH pathogenesis through multiple mechanisms.

In addition to RAGE, the liver expresses various other AGE receptors including the AGE-receptor 1 (AGE-R1) and galectin-3 (Gal-3)^25^. Gal-3 has been associated with inflammation and liver fibrosis and its inhibition was able to reduce fibrosis in preclinical studies of NASH^26,27^, although in a very recent clinical study the efficacy of Gal-3 inhibition has not been confirmed in human patients with NASH, cirrhosis and portal hypertension^28^. Conversely, AGE-R1 is thought to be responsible for the detoxification and clearance of AGEs and for inhibition of AGE-RAGE-induced pro-inflammatory signalling and oxidative stress^29–31^. A decline in the expression of AGE-R1 has been demonstrated to be associated with increases in plasma AGEs in both murine models and diabetic patients^30,32^ and recently also in a RAGE-dependent manner in murine NASH and in the livers of human NASH patients^23^.

In physiological conditions accumulation of AGEs in cells is also prevented by the activity of glyoxalases, the main AGE-detoxifying system acting to degrade methylglyoxal, a major precursor of AGEs, to d-lactate. In the glyoxalase system the glyoxalase-1 (Glo-1) represents the rate limiting step depending on the availability of reduced glutathione (GSH) recruited as cofactor^33^. Impairment of Glo-1 detoxifying potential has been suggested to contribute to the AGEs-mediated tissue damage^33,34^.

Thus, the role of RAGE in the development of NAFLD and NASH and the reciprocal relationships between RAGE and the other AGE-receptors and AGE-detoxifying systems in the liver needs further clarification.

In this study we aimed to analyse the impact of obesity-driven NAFLD on hepatic AGEs accumulation and on the expression of AGEs-binding receptors and detoxifying systems in the liver, and the role of RAGE in modulating NAFLD, hepatic AGEs and the AGE-receptors and AGE-detoxifying systems.

## Results

### RAGE deletion does not reduce AGEs in the liver of LeptrDb−/− mice

For our aims, we used a genetically-obese mouse model, *i.e.* mice lacking the leptin receptor (LeptrDb−/−), with or without additional deletion of RAGE (RAGE+/+LeptrDb−/−, RAGE−/− LeptrDb−/−), compared to wild type lean mice (RAGE+/+LeptrDb+/+). At 13 weeks of age, obese LeptrDb−/− animals displayed increased body and impaired glucose tolerance compared to lean LeptrDb+/+ controls. Although RAGE deletion ameliorated glucose homeostasis and systemic inflammatory markers in obese LeptrDb−/− animals, it did not affect body weight (Supplementary Table S3), as previously described^5^.

**Figure 1:**
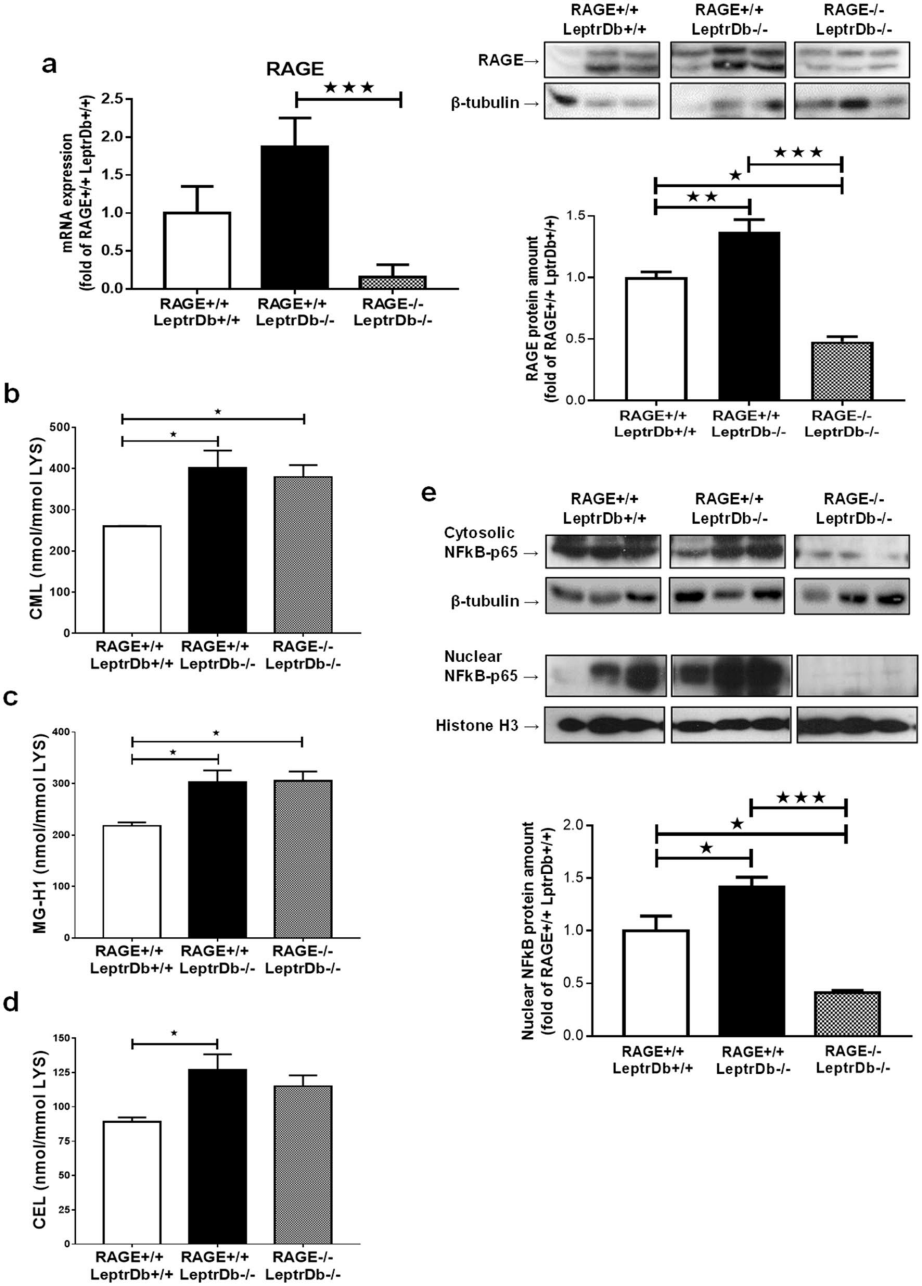
Receptor for advanced glycation endproducts (RAGE) expression and advanced glycation endproducts (AGEs) accumulation in liver. **(a)** PCR and Western blotting analysis for RAGE in liver mRNA and protein extracts from lean controls (RAGE+/+LeptrDb+/+), obese (RAGE+/+LeptrDb−/−), and RAGE KO obese (RAGE−/− LeptrDb−/−) mice. (**b-d**) Mass spectrometry analysis for carboxymethyllysine (CML) (**b**), methylglyoxal 5-hydro-5-methylimidazolones (MG-H1) (**c**), and carboxyethyllysine (CEL) (**d**) in liver lysates. (**e**) Western blotting analysis for cytosolic and nuclear levels of NFkBp65. Results are normalized for the lysine content. Data are means ± SEM of 4–6 animals per group. Statistical significance: *P < 0.05; **P < 0.01; ***P < 0.001.

PCR and western blotting analysis showed increased RNA expression and protein levels of RAGE in the liver of obese (RAGE+/+LeptrDb−/−) mice which were both effectively downregulated when RAGE gene was knocked down (Fig. 1a). In the liver of obese mice we found increased levels of different classes of AGEs: CML (Fig. 1b), methylglyoxal 5-hydro-5-methylimidazolones (MG-H1) (Fig. 1c), and carboxyethyllysine (CEL) (Fig. 1d), measured with state-of-the art UPLC-MSMS, as compared to lean control mice, which were unaffected by RAGE deletion (Fig. 1b–d).

The protein level and nuclear translocation of the pro-inflammatory transcription factor nuclear factor-kappa B (NFkBp65), one of the main targets of RAGE signalling, were increased in the obese RAGE+/+LeptrDb−/− mice, while both cytosolic and nuclear protein levels were completely abrogated in obese RAGE−/− LeptrDb−/− (Fig. 1e).

### RAGE deletion does not prevent steatosis in the liver of LeptrDb−/− mice

**Figure 2:**
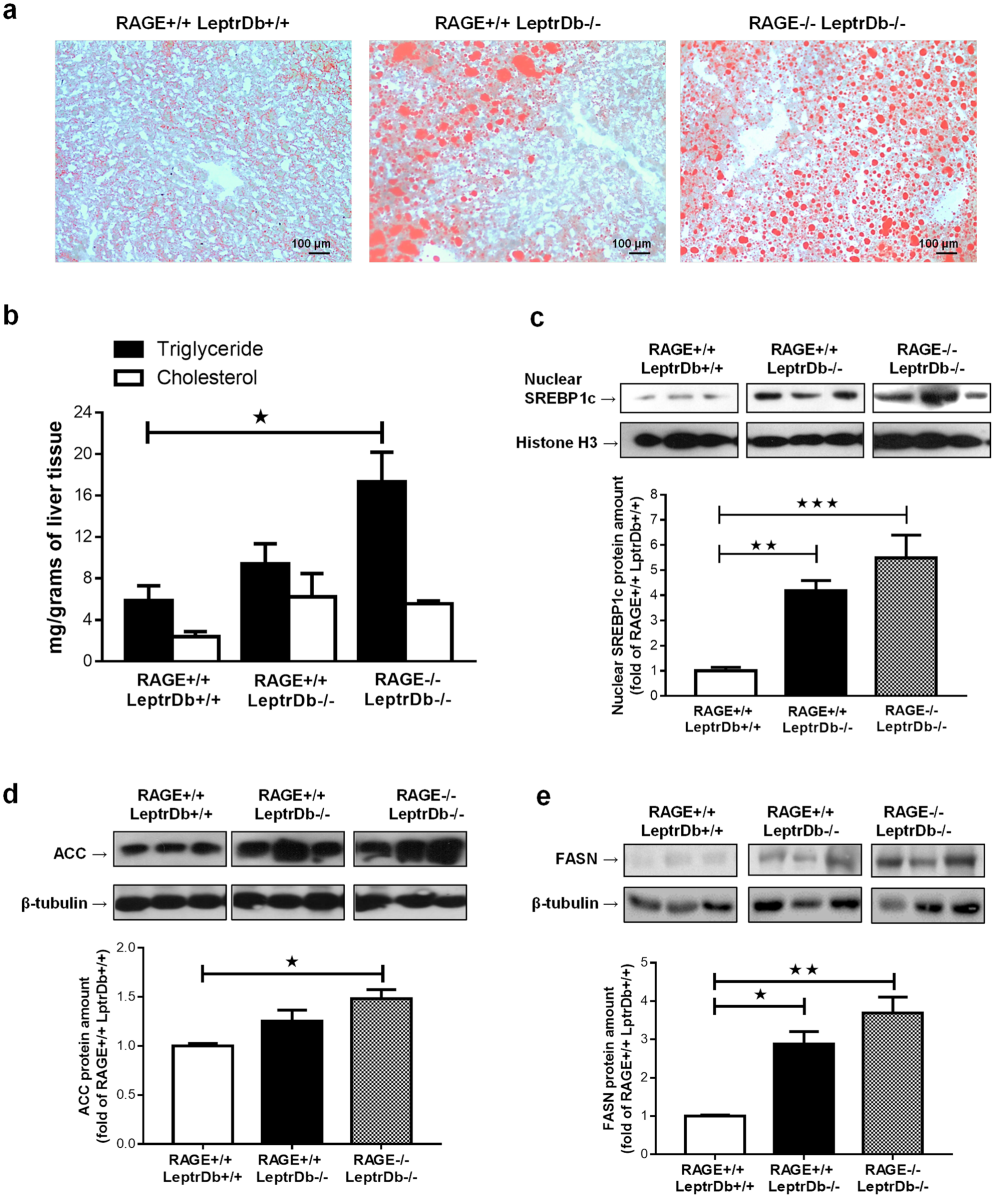
Hepatosteatosis. (**a**) Oil red O staining of 7 µm cryostatic sections of the liver from lean controls (RAGE+/+LeptrDb+/+), obese (RAGE+/+LeptrDb−/−), and RAGE KO obese (RAGE−/− LeptrDb−/−) mice showing hepatic lipids accumulation (20 × magnification). (**b**) Triglyceride and total cholesterol quantification in liver homogenates. (**c**) Western blotting analysis for the sterol regulatory element binding protein 1c (SREBP-1c) in nuclear extracts showing hepatic activation of lipogenesis. (**d,e**) Western blotting analysis for acetyl coenzyme A carboxylase (ACC) (**d**) and for fatty acids synthase (FASN) (**e)** in liver extracts. Data are means ± SEM of 4–6 animals per group. Statistical significance: *P < 0.05; **P < 0.01; ***P < 0.001.

Obese mice showed a marked focal macrovesicular steatosis. RAGE deletion did not prevent total hepatosteatosis in obese animals but caused a more diffuse microvescicular lipid droplet accumulation (Fig. 2a). Lipid accumulation was quantified by analysis of triglycerides and total cholesterol content in liver homogenates (Fig. 2b) showing that obesity-induced accumulation of triglycerides is further enhanced in the absence of RAGE. We next determined the key proteins involved in the development of hepatic steatosis. In the liver of the obese LeptrDb−/− mice we found increased nuclear translocation of the sterol regulatory element binding protein-1c (SREBP1c) (Fig. 2c) and increased expression of the de novo lipid synthesis enzymes acetyl coenzyme A carboxylase (ACC) (Fig. 2d) and fatty acid synthase (FASN) (Fig. 2e), compared to the lean LeptrDb+/+ mice, which tended to be even further increased in obese RAGE−/−LeptrDb−/− mice (Fig. 2c–e).

### RAGE deletion affects the expression of the AGEs-receptors AGE-R1 and Gal-3 in the liver of LeptrDb−/− mice

**Figure 3:**
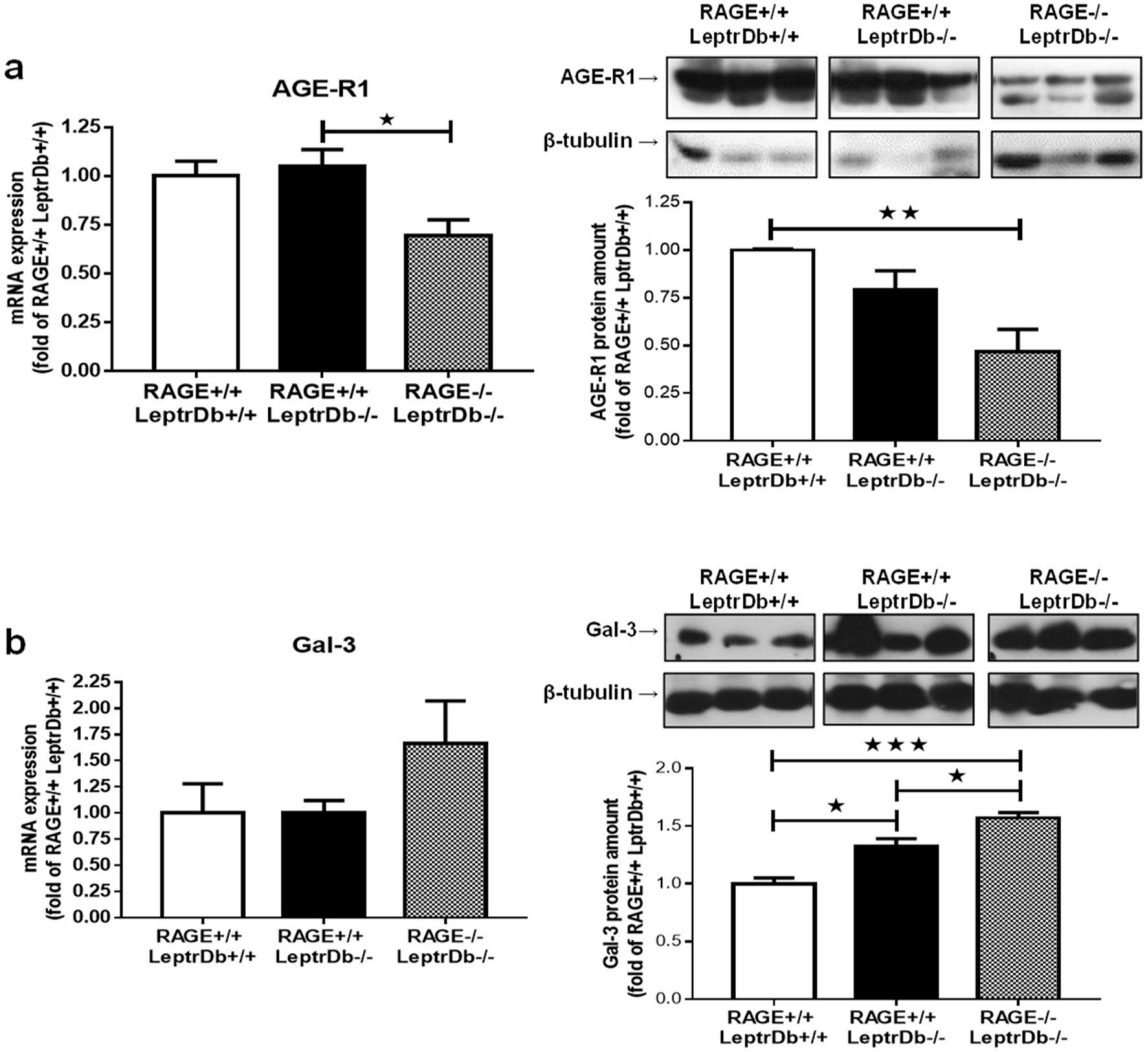
AGEs receptors. PCR and Western blotting analysis for AGE-receptor 1 (AGE-R1) (**a**) and galectin-3 (Gal-3) (**b**) in liver mRNA and protein extracts from lean controls (RAGE+/+LeptrDb+/+), obese (RAGE+/+LeptrDb−/−), and RAGE KO obese (RAGE−/− LeptrDb−/−) mice. Data are means ± SEM of 4–6 animals per group. Statistical significance: *P < 0.05; **P < 0.01; ***P < 0.001.

We next determined the expression of other receptors for AGEs in the liver. Obesity per se did not exert any effect on the gene and protein expression of the detoxifying AGE-receptor AGE-R1 compared to the lean LeptrDb+/+ mice, while RAGE-deficient LeptrDb−/− mice displayed reduced AGE-R1 expression (Fig. 3a).

Obese RAGE+/+LeptrDb−/− mice had significantly increased protein levels of the AGE-receptor Gal-3 in the liver, although no changes were seen at mRNA level, which was further exacerbated in RAGE−/−LeptrDb−/− mice in parallel with significantly increased gene expression (Fig. 3b).

### RAGE deletion impairs the AGEs detoxifying enzymes Glo-1 and aldose reductase in the liver of LeptrDb−/− mice

**Figure 4:**
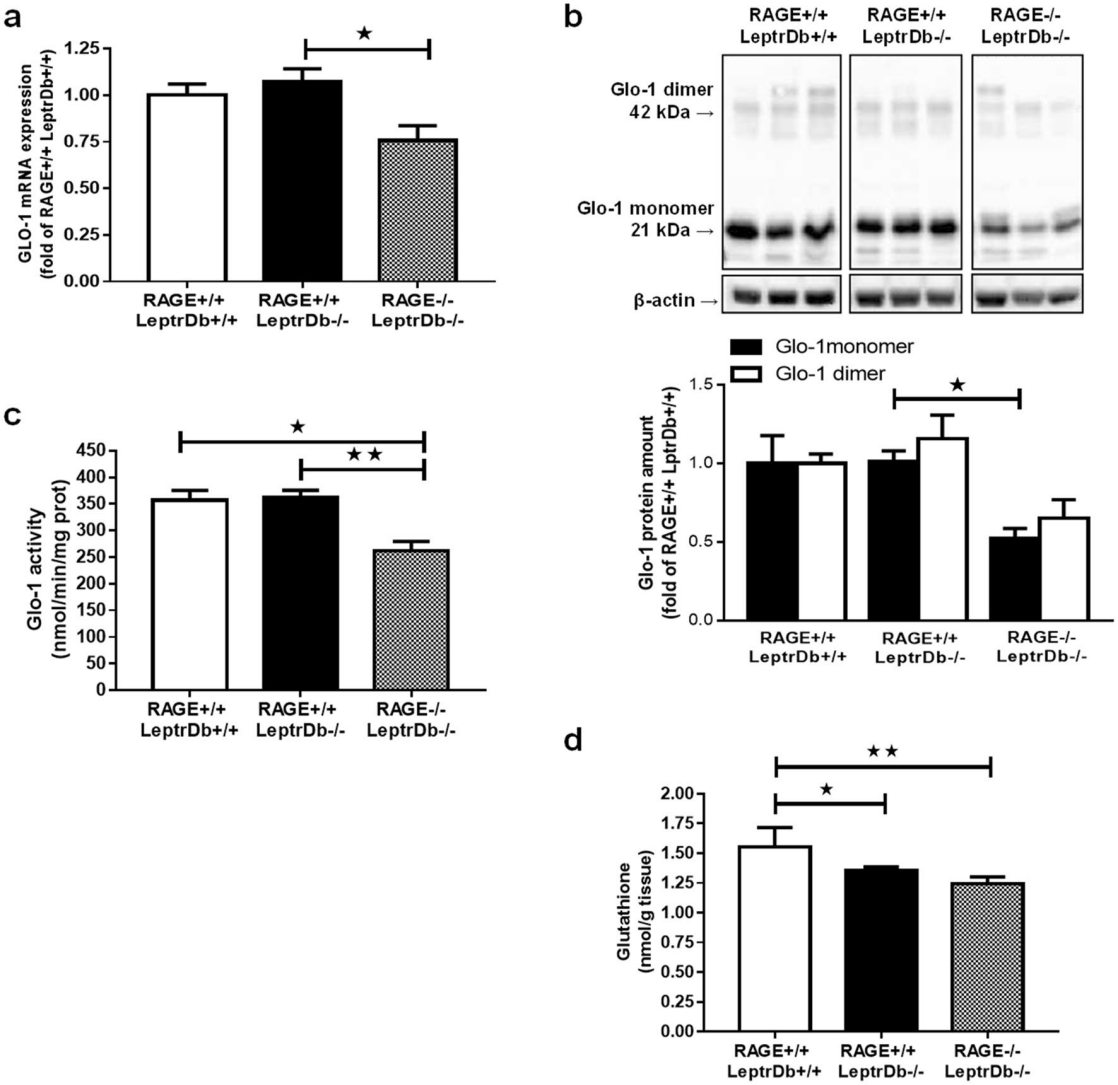
Glyoxalase-1 (Glo-1) expression and activity and glutathione levels. (**a**) PCR analysis for gene expression of Glo-1 in liver from lean controls (RAGE+/+LeptrDb+/+), obese (RAGE+/+LeptrDb−/−), and RAGE KO obese (RAGE−/− LeptrDb−/−) mice. (**b**) Western blotting analysis for Glo-1 protein levels in liver extracts. Histograms report the densitometric analysis normalized for β-actin content. (**c**) Glo-1 enzymatic activity detected in liver lysates. (**d**) Total glutathione in liver lysates. Data are means ± SEM of 4–6 animals per group. Statistical significance: *P < 0.05; **P < 0.01; ***P < 0.001.

Hepatic gene (Fig. 4a) and protein (Fig. 4b) expression, as well as enzymatic activity (Fig. 4c) of Glo-1, were not modified in obese RAGE+/+LeptrDb−/− compared to lean LeptrDb+/+ mice. Remarkably, RAGE-deficiency induced a downregulation of Glo-1 mRNA and protein (Fig. 4a,b). These changes were paralleled by impaired hepatic Glo-1 enzymatic activity (Fig. 4c), suggesting reduced detoxification of methylglyoxal in the liver of obese animals. In addition, we found that total glutathione, an essential cofactor for Glo-1, was reduced in obese animals compared to lean controls, independently of RAGE deletion (Fig. 4d).

In addition, the expression of aldose reductase, another enzyme described to be involved in the detoxification of methylglyoxal, was reduced by obesity in both LeptrDb−/− groups without any modulation by RAGE deletion (Supplementary Fig. S1).

### Different impact of RAGE deletion on the stress-activated pro-inflammatory pathways Nrf2 and NLRP3 inflammasome in the liver of LeptrDb−/− mice

**Figure 5:**
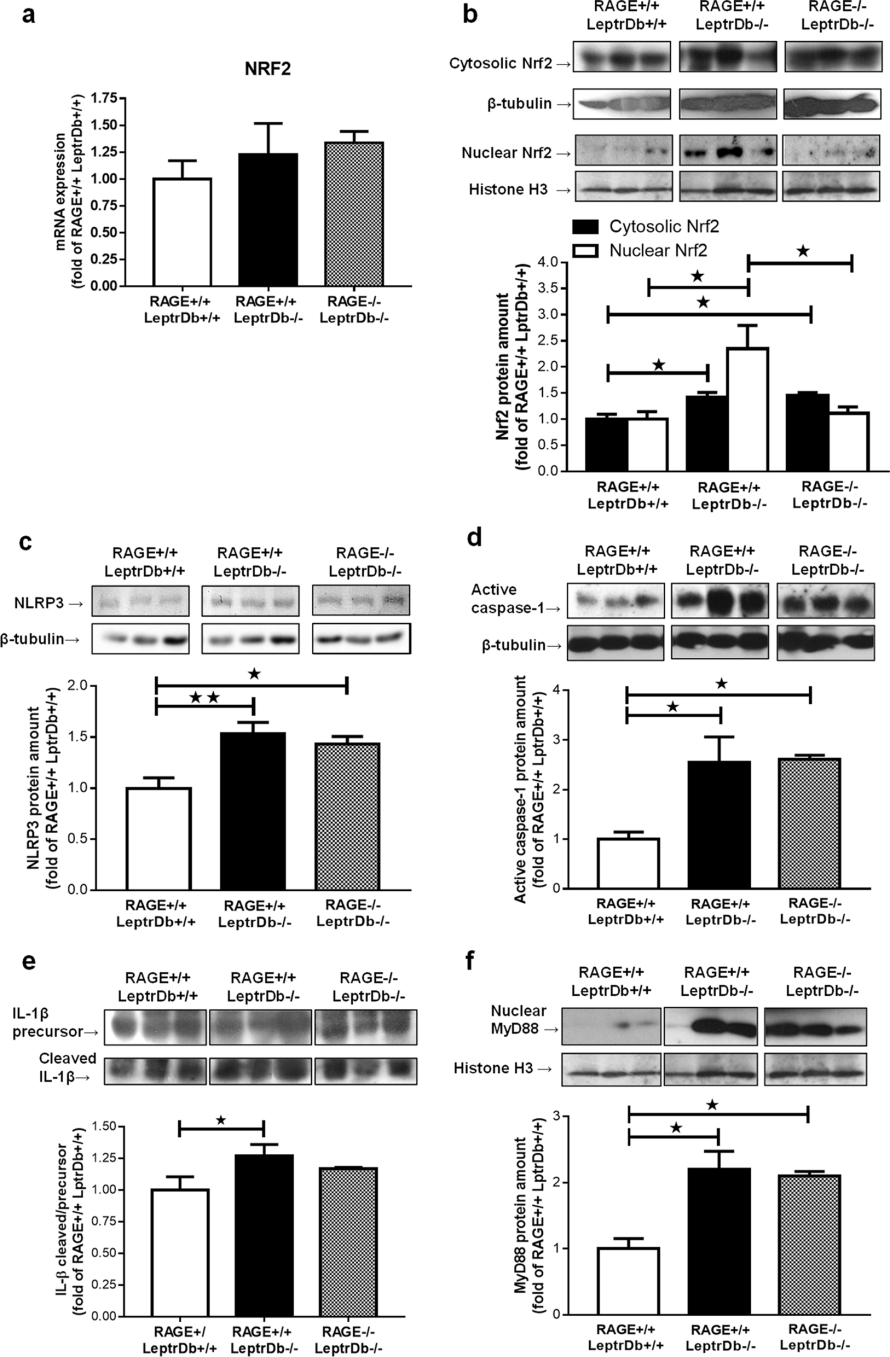
Hepatic inflammation. (**a**) PCR analysis for gene expression of the nuclear factor erythroid 2-related factor 2 (Nrf2) in liver mRNA extracts. (**b**) Western blotting analysis for protein levels of Nrf2 in cytosolic and nuclear extracts of mice liver showing nuclear translocation. (**c–f**) Western blotting analysis for protein levels of the NLR family pyrin domain containing 3 (NLRP3) (**c**), active-caspase-1 (**d**), pro- and cleaved-intereleukin-1β (IL-1β) (**e**), myeloid differentiation primary response 88 (MyD88) (**f**) in liver protein extracts. Data are means ± SEM of 4–6 animals per group. Statistical significance: *P < 0.05; **P < 0.01; ***P < 0.001.

Next, we analysed the activation of the nuclear factor erythroid 2-related factor 2 (Nrf2). This transcription factor is important for the control of AGE-R1 expression and for Glo-1 expression and activity, since it directly regulates the expression of GLO-1, but also regulates different reduced glutathione recycling enzymes that provide GSH to Glo-1 as co-factor for its enzymatic activity. Although mRNA expression of Nrf2 (Fig. 5a) was unaffected in obese RAGE+/+LeptrDb−/− and RAGE−/− LeptrDb−/− mice, nuclear protein levels of Nrf2 were increased in obese RAGE+/+LeptrDb−/− mice, suggesting enhanced transcriptional activity in obesity. This increase was completely absent in obese RAGE−/− LeptrDb−/− animals (Fig. 5b).

Finally, activation of the NLR family pyrin domain containing 3 (NLRP3) inflammasome complex was increased in the liver of the obese mice compared to lean controls, but it was not affected by RAGE deletion, demonstrated by the protein expression of NLRP3 (Fig. 5c), and by expression and cleavage activation of caspase-1 (Fig. 5d) and interleukin-1β (IL-1β) (Fig. 5e). Since the main activator of the NLRP3 platform is NFkB, which activation is completely inhibited by the lacking of RAGE in our obese RAGE−/− mice, we analysed the expression of myeloid differentiation primary response 88 (MyD88), an inducer of NLRP3 priming and activation alternative to NFkB. Although MyD88 was found to be markedly increased in the obese LeptrDb−/− groups, MyD88 was not affected by RAGE deletion (Fig. 5f).

## Discussion

**Figure 6:**
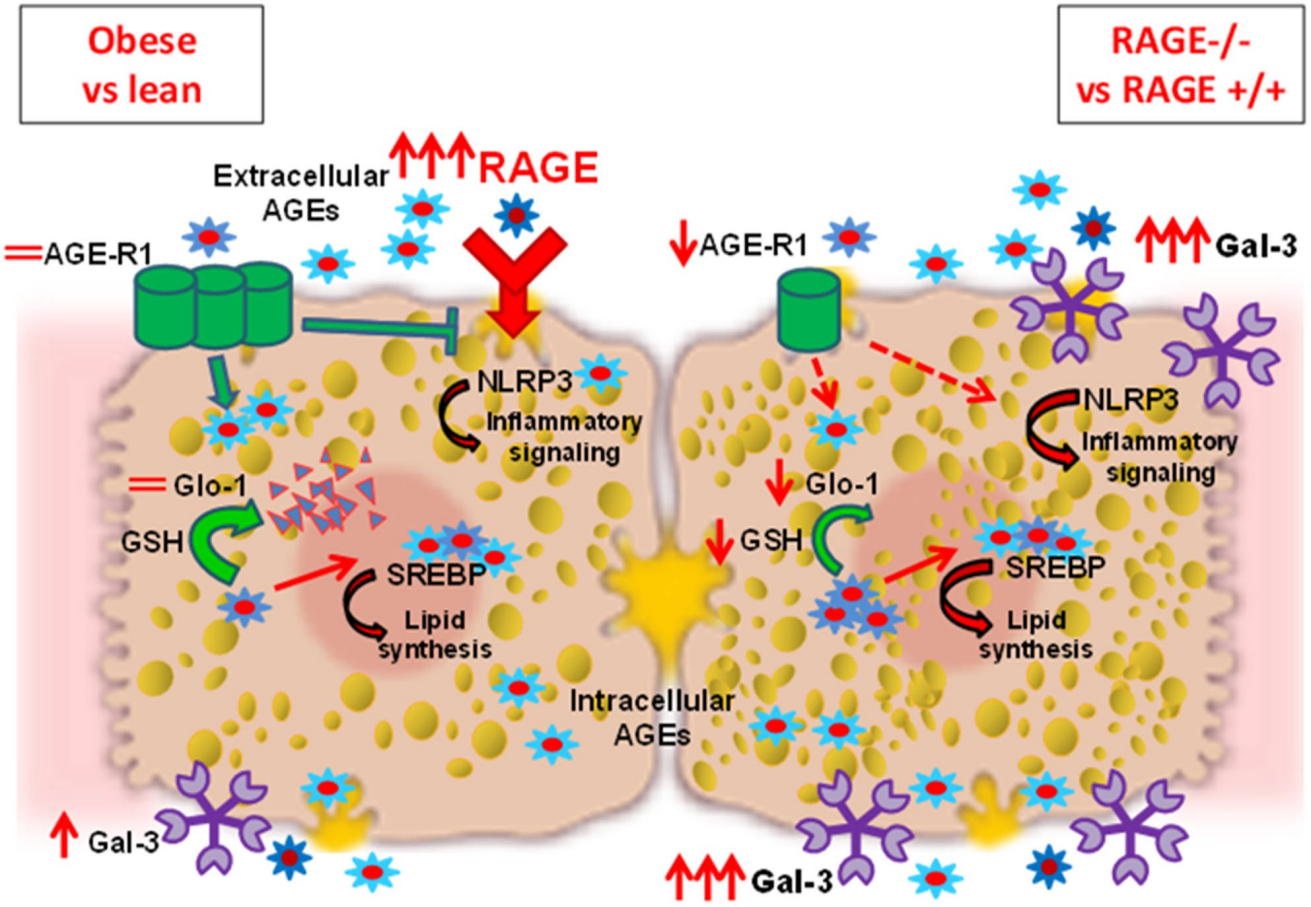
In healthy conditions, physiological levels of circulating AGEs bind to RAGE in the liver evoking a pro-inflammatory signaling. This effect is counteracted by the AGE-receptor-1 (AGE-R1) that competes with RAGE for AGEs binding and causes internalization and degradation of AGEs. The formation of endogenous AGEs is also inhibited by glyoxalase-1 (Glo-1) which detoxifies the AGE precursor methylglyoxal using reduced glutathione (GSH) as cofactor. In the liver of obese mice (RAGE+/+LeptrDb−/−) (left side of image) AGEs are produced in larger amounts and accumulate intracellularly. The expression of RAGE is greatly increased leading to activation of the NLRP3 inflammatory pathway. The presence of the alternative AGE receptor Gal-3 is enhanced, but the detoxifying systems are not induced. The excess of intracellular AGEs activates the de novo lipid synthesis by interfering with the activity of the sterol regulatory element binding protein 1c (SREBP1c), contributing to hepatosteatosis. When RAGE is deleted in the obese background (RAGE−/− LeptrDb−/−) (right side of image), expression of galectin-3 (Gal-3) is further increased and is likely to be involved in a compensative AGEs trapping from the circulation and AGEs-induced NLRP3 inflammatory signalling. The lacking of RAGE is also associated in the liver with reduced AGE-R1 expression and Glo-1 activity that fail to detoxify intracellular AGEs, thus contributing to the maintenance of high hepatic AGEs levels, and prolonging SREBP1c activation and lipids accumulation. Thus, the deletion of RAGE fails to prevent AGE accumulation, hepatosteatosis and inflammation in obese mice, due to alterations of other AGE-receptors and impairment of AGE-detoxifying system.

In this study we show in an animal model of obesity-induced NAFLD increased levels of hepatic AGEs, of RAGE expression and of Gal-3 protein amount, with no relevant modifications of other AGEs receptors and detoxification systems. Interestingly, deletion of RAGE neither prevented NAFLD development nor altered the accumulation of hepatic AGEs. In addition, RAGE deletion resulted in reduced AGE-R1 and Glo-1 and further increased Gal-3 receptor in the liver (Fig. 6). This reduced AGE-detoxifying potential and increased Gal-3 are most likely involved in the persistent hepatosteatosis and inflammation when RAGE is depleted and should be taken into account when investigating the role of the AGE-RAGE axis in liver pathologies.

A number of studies has shown that RAGE plays a role in toxic, ischaemic, and cholestatic liver damage^35–37^. Neutralizing RAGE with specific antibodies has been reported to prevent hepatic stellate cells activation and fibrogenesis in a murine model of carbon tetrachloride-induced liver fibrosis^38^. Moreover, blocking RAGE in hepatic stellate cells prevented the increase of AGEs-induced inflammatory and oxidative stress markers^39^. Additionally, both systemic treatment with pyridoxamine and hepatocyte-specific RAGE deletion were shown to prevent NASH in a model relying on a high AGEs diet^23^. In our study, we used mice with a homozygous mutation in the leptin receptor (LeptrDb−/−) which are hyperphagic and are considered a good model of obesity and diabetes induced by excessive calories intake. The intrahepatic accumulation of MG-H1, CML, and CEL in the obese LeptrDb−/− mice were accompanied by increased expression of RAGE, activation of NFkB, and to activation of the SREBP1c lipogenic pathway, probably contributing to hepatosteatosis.

In accordance, it has been reported that CML accumulation in the liver of obese individuals is associated to hepatic pro-inflammatory genes expression and correlates with the grade of steatosis and steatohepatitis^20^. The development of steatosis may have contributed to hepatic AGEs accumulation since FFAs stimulate CML accumulation in hepatocytes and subsequently elicit inflammatory reactions via RAGE^20^. Although we found that the genetic deletion of RAGE is effective in lowering NFkB in the liver, it did not improve hepatic steatosis. In accordance, the genetic deletion of RAGE in the hyperlipidemic Ldlr−/− mouse model fed a western diet did not improve hepatosteatosis, nor systemic and hepatic inflammation, and did not reduce plasma and liver AGEs^22^. Surprisingly, hepatocyte-specific deletion of RAGE was recently reported to reduce hepatic AGEs, liver inflammation and steatosis elicited by a diet rich in AGEs^23^. It is known that RAGE is involved in trapping of CML selectively in adipose tissue^5^ and that its deletion leads to reduced adipocyte size, adipose tissue expansion, and inflammation^40,41^. Whole-body deletion of RAGE may thus lead to a dysbalance in the body’s AGEs levels due to the loss of trapping in adipose tissue, while hepatocyte-specific deletion would still allow trapping of circulating AGEs by RAGE in adipose tissue. Thus, the persistence of hepatosteatosis and inflammation in the liver of the LeptrDb−/− animals in spite of the absence of RAGE may, at least partially, be explained by a persistently high level of AGEs in the organism.

It has been reported that uncontrolled increase of AGEs leads to hyperexpression of RAGE with concomitant depletion of AGE-R1 expression and function^23,29,30,42–44^, while induction of AGE-R1 overexpression has a protective effect on both AGEs accumulation and RAGE hyperexpression^27,31,44^. Specifically, a recent study has suggested that in high-AGEs diet-induced NASH, increased RAGE downregulates the expression of AGE-R1 gene through Nrf2 suppression^23^. In the present study, in the liver of LeptrDb−/− mice, we found that the accumulation of AGEs and the increased RAGE levels found in obese mice were not associated with a change in AGE-R1 levels. This difference is possibly due to the different impact that the AGEs-diet exert on AGE receptors in comparison to our model of exceeding calories intake-induced NAFLD. However, the genetic deletion of RAGE in our obese model concomitantly led to a significant downregulation of AGE-R1 via a hereto unknown mechanism, which may counteract any beneficial effects of RAGE-deficiency.

In addition, Gal-3 is another important ubiquitously distributed high affinity receptor for AGEs involved in removal of AGEs from plasma leading to cell activation^45,46^. Non-consistent effects have been reported for Gal-3 knockout in the liver, with either an improvement or a worsening of steatosis and liver damage^47,48^. Although Gal-3 expression was only slightly increased in the obese LeptrDb−/− model compared to the lean Leptr+/+ model, RAGE-deficient obese animals showed a strong, possibly compensatory, increase of Gal-3 expression.

Furthermore, Gal-3 has been previously involved in the activation of the NLRP3 inflammasome pathway, contributing to inflammatory response^49^. Accordingly, studies in the liver of Gal-3 knockout mice indicate attenuation of inflammation, hepatocyte injury and fibrosis, and insulin resistance^26,47^. In our model the consistent increased gene and protein expression of Gal-3 in the RAGE−/−LeptrDb−/− mice was accompanied by sustained NLRP3 inflammasome pathway activation and this was paralleled by markedly increased expression of MyD88, an alternative inducer of NLRP3 complex to the classical NFkB pathway, whose activation was indeed completely suppressed by the RAGE deletion^50^. Moreover, the here observed progressive increase of Gal-3 protein level across the experimental groups seems to positively correlate with the activation of the lipogenic SREBP1c pathway, with the triglyceride content, and with the grade of steatosis, as indicated by some papers on NAFLD and NASH in animal models and by a clinical study on liver biopsies from NAFLD children^51–53^. Thus, Gal-3 is likely to be a good candidate for the compensative trapping of circulating AGEs in the liver of obese mice in absence of RAGE, enhancing AGEs-induced liver damage.

On the other hand, the rising AGE levels can be counteracted by the detoxifying activity of Glo-1^54^. In animal models of genetically induced obesity variable alterations of Glo-1 gene and protein expression have been described depending on the experimental conditions and the examined tissue^55,56^, with generally a decrease in enzymatic activity that can rather be due to due to the high AGEs influx and to the exhaustion of its cofactor GSH^57,58^. Indeed, the activation of RAGE leads to inflammation and oxidative stress with consequent depletion of the antioxidant tripeptide GSH. In the liver of our obese LeptrDb−/− mice, no alterations in gene or protein expression, neither in enzymatic activity of Glo-1 was detected in comparison to the lean controls, but deletion of RAGE in the LeptrDb−/− background significantly impaired Glo-1 gene expression with consequent reduced protein levels and compromised activity. Notably, gene expression of Glo-1 is transcriptionally regulated by the transcription factor Nrf2 which also controls the expression of enzymes responsible for the GSH synthesis and recycling. In our model, the nuclear protein levels of Nrf2 were enhanced in the obese LeptrDb−/− mice, but this increase was completely prevented when RAGE was deleted. This impaired nuclear translocation of Nrf2 in absence of RAGE might account for the reduced Glo-1 expression and GSH levels and consequent reduced AGEs-detoxifying activity. In addition, in the obese LeptrDb−/− groups of mice, we also observed significant reduction in the expression of aldose reductase, an enzyme involved in the metabolism of glucose that catalyzes the reduction of AGEs precursors and regulate the expression of RAGE^59^, with no additional alteration when RAGE was deleted.

This study has some limitations. First, we do not provide evidence for a direct causal role of AGEs in induction of SREBP1c and NLRP3 pathways. However, we and others have demonstrated elsewhere that inhibition of AGEs production or blocking of AGE/RAGE signalling reverses both SREBP1c and NLRP3 upregulation, concomitantly improving steatosis and inflammation^43,60–63^.

Second, although we report a clear association between the genetic deletion of RAGE and modifications of the AGE-detoxifying systems and AGE-receptors, this model does not allow us to disentangle whether these compensatory mechanisms are only at play when RAGE is deactivated before birth, or whether such compensation could also be relevant in a more acute way of inactivating RAGE.

In conclusion, despite the lacking of RAGE in the liver of LeptrDb−/− mice, high levels of intrahepatic AGEs were maintained, possibly caused by both a compensatory trapping exerted by Gal-3 and a reduced detoxifying potential due to AGE-R1, Glo-1, and aldose reductase impairment. As a consequence, hyperactivation of lipogenesis, which has been previously demonstrated to be directly activated by AGEs^21,43^, was enhanced, while the increased expression of Gal-3 contributed to both the persistent NLRP3 inflammasome activation, not depending on NFkB, and steatosis.

Our results confirm that AGEs might be involved in the development of NAFLD through different mechanisms, involving either direct AGEs effects or various receptor-mediated signalling. However, RAGE targeting does not seem to be effective in the prevention of NAFLD in conditions of obesity since its deletion influences other AGE-receptors and AGE-detoxifying systems, indirectly demonstrating a complex tissue specific interaction of these molecules.

In perspective, trying to deeply understand this reciprocal control mechanism among AGEs receptors and detoxifying systems will allow to identify molecular targets and therapeutic tools to modulate and prevent the pathogenic contribution of AGEs to hepatic metabolic disturbances.

## Materials and methods

### Animals

We analysed the livers of mice previously used for another study on adipose tissue. Physiological parameters and methods are reported in Gaens et al.^5^. Briefly, obese RAGE-deficient mice (RAGE−/− LeptrDb−/−) were generated by crossing obese, non-insulin dependent diabetic C57BLKS-LeptrDb (LeptrDb−/−) (Charles River, Maastricht, the Netherlands) with RAGE−/− mice (obtained from Heidelberg University) for several generations. All mice were fed ad libitum with a standard control diet (catalog #D11112201; Research Diets Inc., New Brunswick, NJ, USA). At 13 weeks of age, lean control C57BLKS (RAGE+/+LeptrDb+/+), obese (RAGE+/+LeptrDb−/−) and their littermate obese RAGE-deficient (RAGE−/− LeptrDb−/−) mice were sacrificed by CO2/O2 inhalation followed by exsanguination via cardiac puncture, plasma was collected and liver was removed and sectioned for following analysis. The animal protocol was approved by the Ethic Committee of Maastricht University. All animal experiments and methods were in compliance with the guidelines from the Directive 2010/63/EU of the European Parliament on the protection of animals used for scientific purposes and follow the ARRIVE guidelines.

### Markers of inflammation

Mouse inflammatory panels for interferon (IFN)-γ, tumor necrosis factor (TNF)-α, IL-10 assays were purchased from Meso Scale Discovery (MSD, MD, USA)^5^. All reagents were provided with the MSD kit, and measurements were performed according to the manufacturer’s instructions.

### mRNA extracts and PCR analysis

Total RNA was extracted from liver samples using TRIzol (Invitrogen, Bleiswijk, the Netherlands), and was reverse transcribed with the iScript cDNA Synthesis Kit (Bio-Rad, Veenendaal, the Netherlands). The expression of target genes was measured quantitatively by real-time PCR using SYBR Green mix (Bioline, London, U.K.). All primer sets used are listed in Supplementary Table S1. mRNA expression levels were normalized to two reference genes (cyclophilin A and β2-microglobulin), and data were analyzed with the ΔCT method^61^. Data are expressed as normalized gene expression levels relative to wild-type RAGE+/+LeptrDb+/+mice.

### Total, nuclear, and cytosolic protein extracts

Total proteins were extracted from 10% (w/v) liver homogenates in RIPA buffer (0.5% Nonidet P-40, 0.5% sodium deoxycholate, 0.1% SDS, 10 mmol/L EDTA, and protease inhibitors). After 40 min of incubation in ice, samples were sonicated and cleared by centrifugation at 15,000×*g* at 4 °C for 40 min. Supernatants were removed and stored at − 80 °C until use^43^.

Cytosolic and nuclear proteins were obtained from livers homogenized at 10% (wt/vol) in a homogenization buffer containing 20 mM HEPES (pH 7.9), 1 mM MgCl2, 0.5 mM EDTA, 1% Nonidet P-40, 1 mM EGTA, 1 mM DTT, 0.5 mM PMSF, 5 μg/mL aprotinin, 2.5 μg/mL leupeptin and 2 mM NaVO_3_. Homogenates were centrifuged at 1000×*g* for 5 min at 4 °C. Supernatants were removed and centrifuged at 105,000×*g* at 4 °C for 40 min to obtain the cytosolic fraction. The pelleted nuclei were resuspended in extraction buffer containing 20 mM HEPES (pH 7.9), 1.5 mM MgCl2, 300 mM NaCl, 0.2 mM EDTA, 20% glycerol, 1 mM EGTA, 1 mM DTT, 0.5 mM PMSF, 5 μg/mL aprotinin, 2.5 μg/mL leupeptin and 2 mM NaVO_3_ and incubated on ice for 30 min for high-salt extraction, followed by centrifugation at 15,000 g for 20 min at 4 °C. The resulting supernatants containing nuclear proteins were carefully removed and stored at − 80 °C^58^.

Protein content was determined using the Bradford assay (Bio-Rad, Hercules, CA, USA).

### Western blotting analysis

Equal amounts of proteins were separated by SDS-PAGE and electrotransferred to nitrocellulose membrane (GE-Healthcare Europe, Milano, Italy). Membranes were probed with primary antibodies, listed in Supplementary Table S2, followed by incubation with appropriated horseradish peroxidase (HRP)-conjugated secondary antibodies (Bio-Rad). Proteins were detected with Clarity Western ECL substrate (Bio-Rad) and quantified by densitometry using analytic software (Image Lab; Bio-Rad; https://www.bio-rad.com/it-it/product/image-lab-software). Results were normalized with respect to densitometric value of mouse anti-β-actin (Santa Cruz Biotechnology) for total and cytosolic extracts and mouse anti-histone H3 (Abcam) for nuclear proteins^43,58^ and then expressed as fold of wild-type RAGE+/+LeptrDb+/+mice value.

### UPLC-MSMS analysis of AGEs

To measure CML, CEL, MG-H1, and lysine, 25 μL of liver homogenates were subjected to a reduction step in 100 mmol/L sodium borohydride to prevent CML neo-formation. Next, samples were deproteinized with trifluoroacetic acid and then hydrolyzed by adding 6 N HCl to the protein pellet and incubated for 20 h at 90 °C. After hydrolysis, 40 μL hydrolysate and 20 μL internal standard were mixed in a reaction vial and evaporated to dryness under nitrogen at 70 °C and derivatized with 100 μL 1-butanol:HCl (3:1, v/v) for 90 min at 70 °C. Samples were then evaporated to dryness under nitrogen and redissolved in 200 μL water. For measurement of lysine, 10 μL hydrolysate was diluted in 800 μL water. Twenty μL of this mixture and 20 μL internal standard was diluted in 500 μL 10 mmol/L ammonia. Derivatized CML, CEL and MG-H1, and underivatized lysine were analyzed by UPLC tandem MS. Protein-bound CML, CEL and MG-H1 sample concentrations were calculated by comparison to calibration curves. All protein-bound fractions of AGEs were expressed per mmol lysine to adjust for the amount of protein per sample^64^.

### Enzymatic activity of Glo-1

Glo-1 activity was measured in total protein liver extracts, according to the method of McLellan et al.^65^. In short, Glo-1 activity was assayed by spectrophotometry (Synergy; BioTek, Winooski, VT), by measuring the increase in absorbance at 240 nm as a result of the formation of S-d-lactoylglutathione for 20 min.

### Lipids accumulation

Neutral lipids accumulation in the liver was evaluated by Oil Red O staining on 7-μm liver cryostatic sections. Stained tissues were viewed under an Olympus Bx4I microscope (× 20 magnification) with an AxioCamMR5 photographic attachment (Zeiss, Gottingen, Germany)^66^. Tissue triglyceride and total cholesterol levels were determined on liver homogenates by standard enzymatic procedures using reagent kits (FAR Diagnostics, Verona, Italy).

### Statistical analysis

The Shapiro–Wilk test was used to assess the normality of the variable distributions. One-way ANOVA followed by Bonferroni's post-hoc test was adopted for comparison among the experimental groups. Data were expressed as mean ± s.e.m. Statistical tests were performed with GraphPad Prism 7.0 software package (GraphPad Software, San Diego, CA, USA; https://www.graphpad.com/scientific-software/prism). Threshold for statistical significance was set to P < 0.05.

### Materials

All compounds were purchased from Sigma Chemical, unless otherwise stated.

## Supplementary Information

Supplementary Information.
