# Supplementary material for: Deletion of RAGE fails to prevent hepatosteatosis in obese mice due to impairment of other AGEs receptors and detoxifying systems
Source: Sci Rep. 2021 Aug 30;11:17373. doi: 10.1038/s41598-021-96859-7 (PMC8405685; doi:10.1038/s41598-021-96859-7)
Supplement: Supplementary file 1 — Supplementary Information. [file 41598_2021_96859_MOESM1_ESM.pdf]

Deletion of RAGE fails to prevent hepatosteatosis in obese mice due to impairment of other AGEs receptors and detoxifying systems.

Wouters et al.

Supplementary Table 1. Primers for RT-PCR

PRIMER	FORWARD	REVERSE
RAGE	5’-GTCCGAGTCTACCAGATTCCT -3’	5’-GAAGTTTCCCATCTAAGTGCC-3’
AGER-1	5’-ACCTTCAAGACCGCAGATGA-3’	5’-CACGTTGATGTTGCCTCCAA-3’
Gal-3	5’-CCCGCATGCTGATCACAATC-3’	5’-GGGGTTAAAGTGGAAGGCAA-3’
Aldose reductase	5’-CGTGATACCTAGTGACACCGA-3’	5’-GCCAGGTTTGTTCAAGATCCT-3’
Glo-1	5'-CCCTGCTATGAAGTTCTCGCTC-3'	5'-GAGCTCAAGGGTGGCTTTTCT-3’
Nrf2	5’-TTGGCAGAGACATTCCCAT-3’	5’-GCTGCCACCGTCACTGGG-3’

Supplementary Table 2. Primary antibodies for western blotting analysis.

ANTIBODIES	COMPANY	CAT. NO.
Mouse monoclonal anti-RAGE	Santa Cruz Biotechnology, Inc., Dallas, TX, USA	sc-365154
Rabbit Polyclonal anti-SREBP1c	Abcam	ab44153
Mouse Monoclonal anti-α-Tubulin	Abcam	ab7291
Mouse Monoclonal anti-Gal-3	Affinity BioReagents, Golden, CO, USA	MA1-940
Rabbit Polyclonal anti-ACC	Cell Signaling Technology, Danvers, MA, USA	3662
Rabbit Monoclonal anti-NLRP3	Epitomics, Burlingame, CA, USA	3560-1
Rabbit Polyclonal anti-Glo-1	Genetex, Irvine, CA, USA	GTX105792
Mouse Monoclonal anti-β-Actin	Santa Cruz Biotechnology	sc-47778
Rabbit Polyclonal anti-Caspase 1 (p10)	Santa Cruz Biotechnology	sc-514
Mouse Monoclonal anti-FASN	Santa Cruz Biotechnology	sc-55580
Rabbit Polyclonal anti-Histone H3	Santa Cruz Biotechnology	sc-10809
Mouse Monoclonal anti-IL-1β	Santa Cruz Biotechnology	sc-52012
Mouse Monoclonal anti-MyD88	Santa Cruz Biotechnology	sc-74532
Mouse Monoclonal anti-NFkB-p65	Santa Cruz Biotechnology.	sc-8008
Mouse Monoclonal anti-OST48 (AGER1)	Santa Cruz Biotechnology	sc-74408
Rabbit Polyclonal anti-Nrf2	Thermo Fisher Scientific, Waltham, MA, USA	PA5-27882

Supplementary Table 3. Body weight and cytokines plasma levels in control (RAGE+/+ LeptrDb+/+), obese (RAGE+/+ LeptrDb-/-), and RAGE KO obese (RAGE-/- LeptrDb-/-) mice. Data are means ± SEM of 5-10 animals per group. Statistical significance: *P<0,05 vs RAGE+/+ LeptrDb+/+; §P<0,05 vs RAGE+/+ LeptrDb-/-.

	RAGE+/+ LeptrDb+/+	RAGE+/+ LeptrDb-/-	RAGE-/- LeptrDb-/-
Body weight (g)	24.1 ± 0.6	35.0 ± 5.0	39.5 ± 5.0
IL-1β (pg/ml)	2.4 ± 0.9	8.0 ± 1.3*	2.4 ± 1.0§
IFN-γ (pg/ml)	0.9 ± 0.1	2.1 ± 0.3*	1.1 ± 0.2§
TNF-α (pg/ml)	1.0 ± 0.4	2.3 ± 0.6*	0.6 ± 0.7§

Supplementary Figure 1. PCR analysis for gene expression of the AGEs detoxyfying enzyme aldose reductase in liver mRNA extracts. Data are means ± s.e.m. of 4-6 animals per group. Statistical significance: **P<0,01; ***P<0,001.

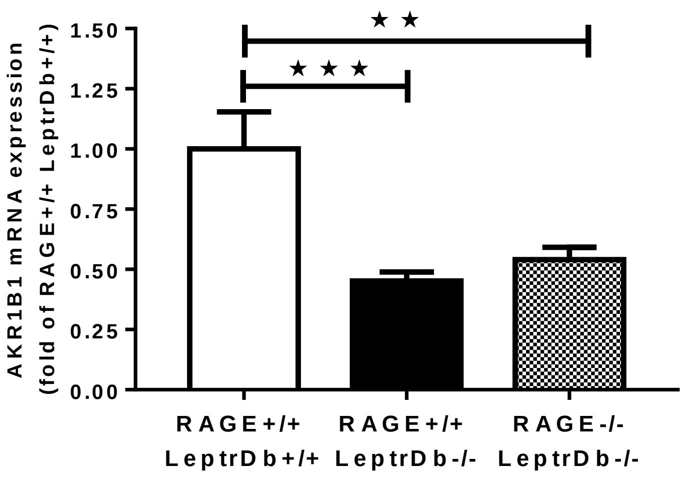

Supporting information

Ponceau S staining

Total extracts

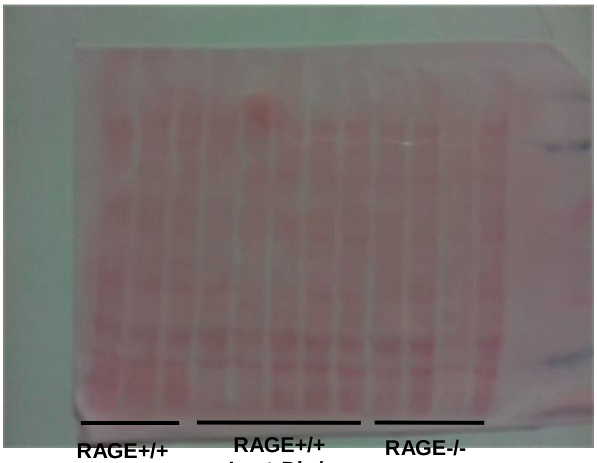

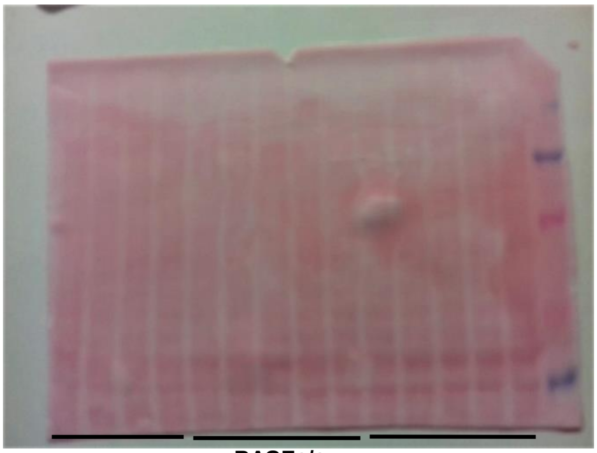

Cytosolic extracts

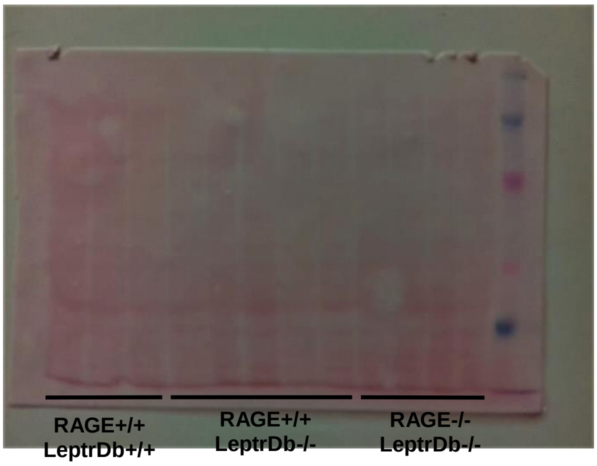

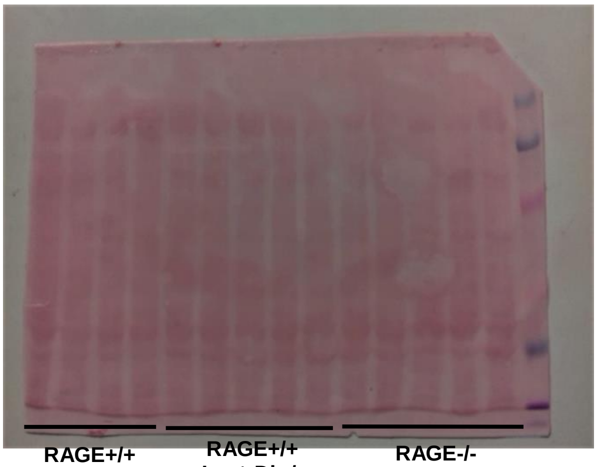

Original blots for western blotting figures

Figure 1.

A

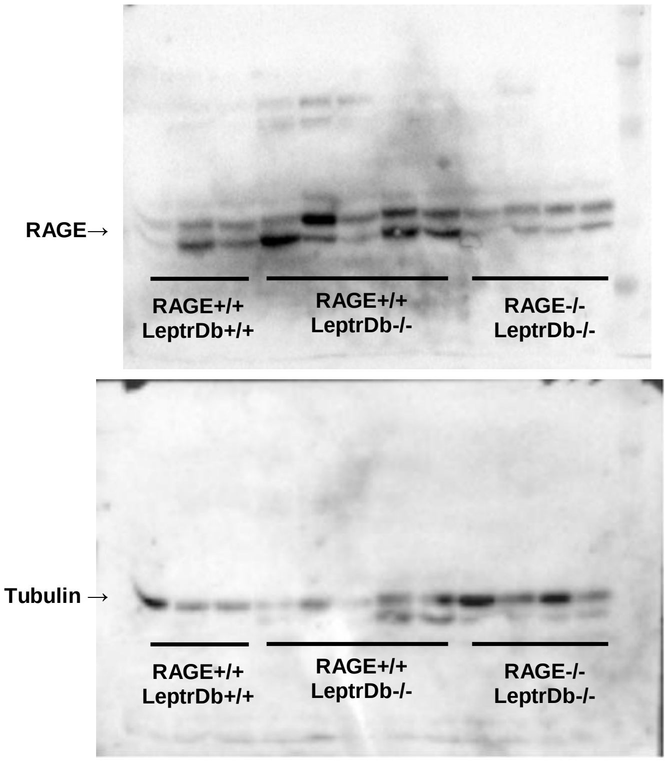

E

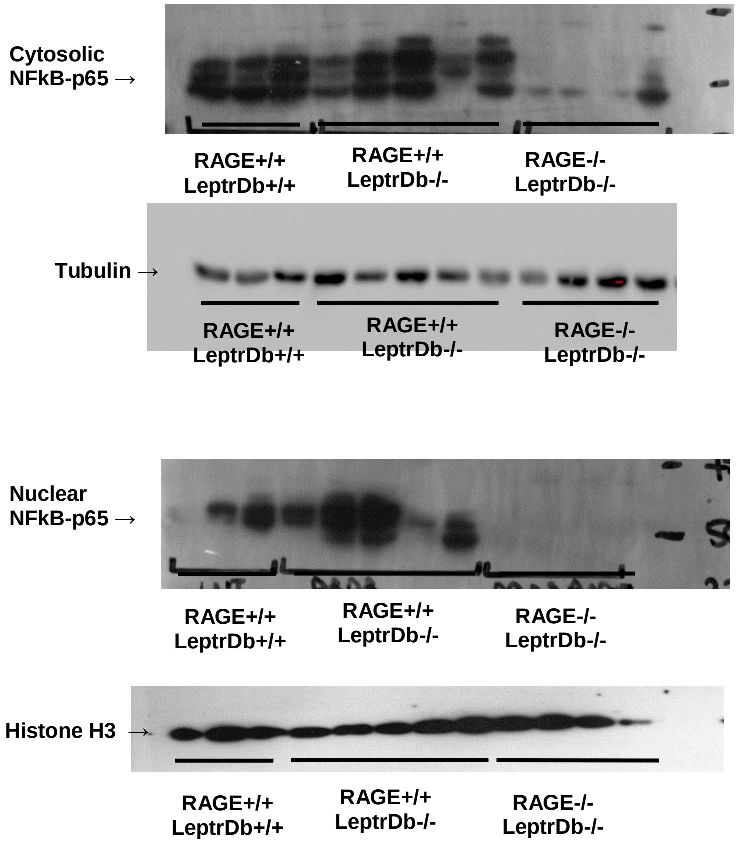

Figure 2.

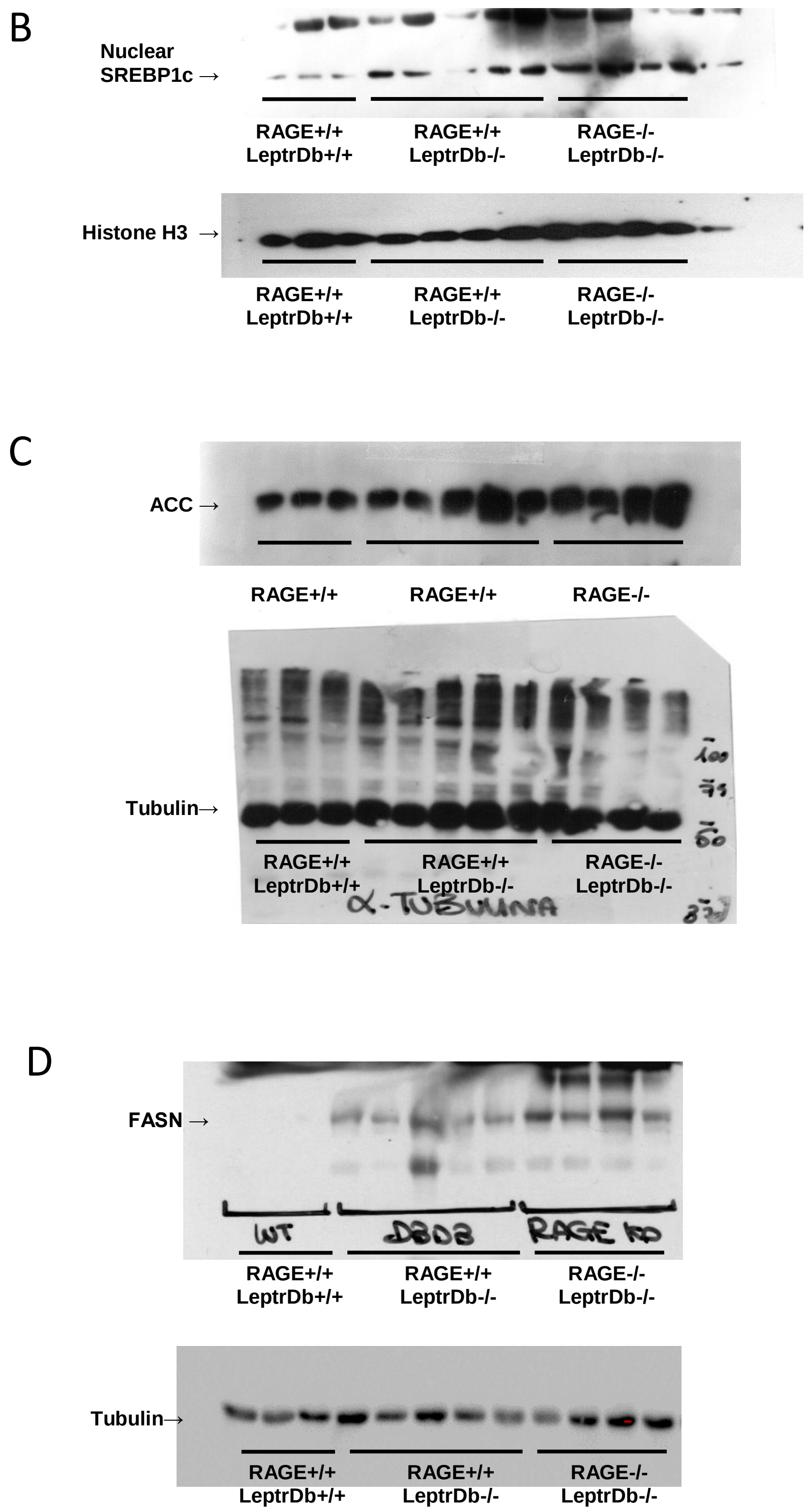
B
Nuclear SREBP1c →
RAGE+/+ LeptrDb+/+
RAGE+/+ LeptrDb-/-
RAGE-/- LeptrDb-/-
Histone H3 →
RAGE+/+ LeptrDb+/+
RAGE+/+ LeptrDb-/-
RAGE-/- LeptrDb-/-
C
ACC →
RAGE+/+
RAGE+/+
RAGE-/-
Tubulin→
100
75
50
RAGE+/+ LeptrDb+/+
RAGE+/+ LeptrDb-/-
RAGE-/- LeptrDb-/-
α-TUBULINA
D
FASN →
WT
DBDB
RAGE KO
RAGE+/+ LeptrDb+/+
RAGE+/+ LeptrDb-/-
RAGE-/- LeptrDb-/-
Tubulin→
RAGE+/+ LeptrDb+/+
RAGE+/+ LeptrDb-/-
RAGE-/- LeptrDb-/-

Figure 3.

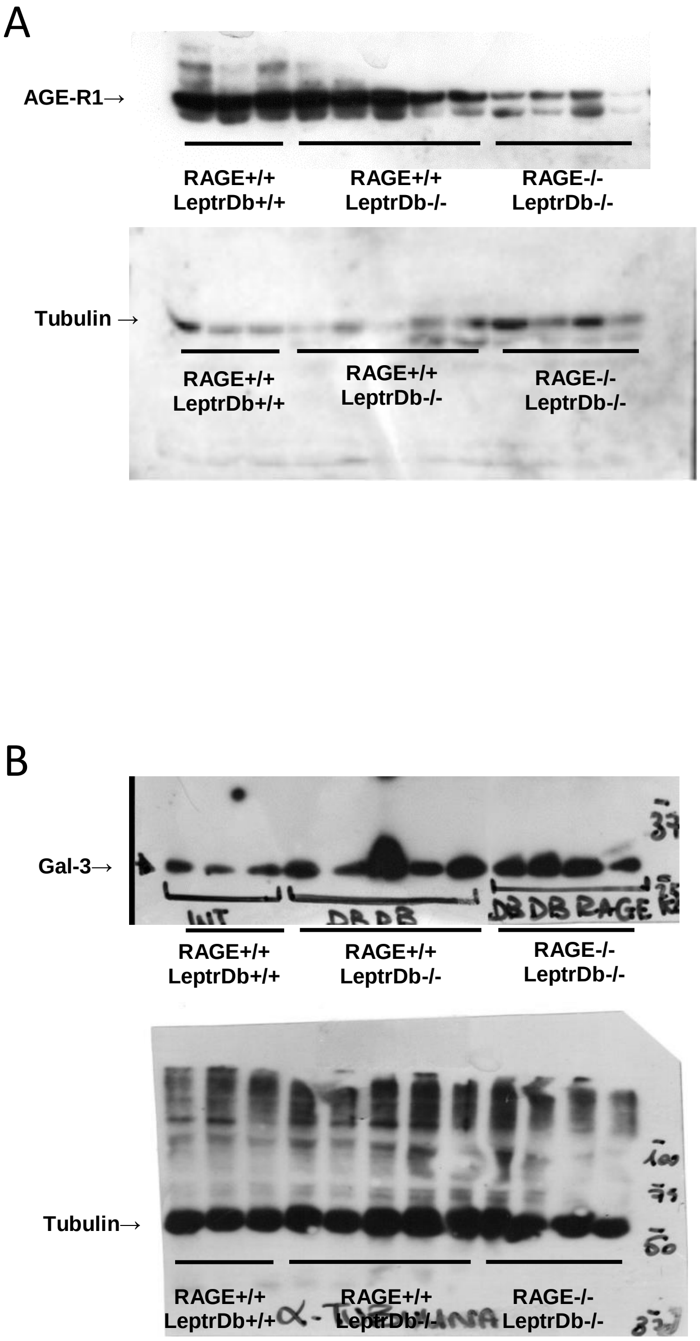
A
AGE-R1→
RAGE+/+ LeptrDb+/+
RAGE+/+ LeptrDb-/-
RAGE-/- LeptrDb-/-
Tubulin →
RAGE+/+ LeptrDb+/+
RAGE+/+ LeptrDb-/-
RAGE-/- LeptrDb-/-
B
Gal-3→
RAGE+/+ LeptrDb+/+
RAGE+/+ LeptrDb-/-
RAGE-/- LeptrDb-/-
Tubulin→
RAGE+/+ LeptrDb+/+
RAGE+/+ LeptrDb-/-
RAGE-/- LeptrDb-/-

Figure 4.

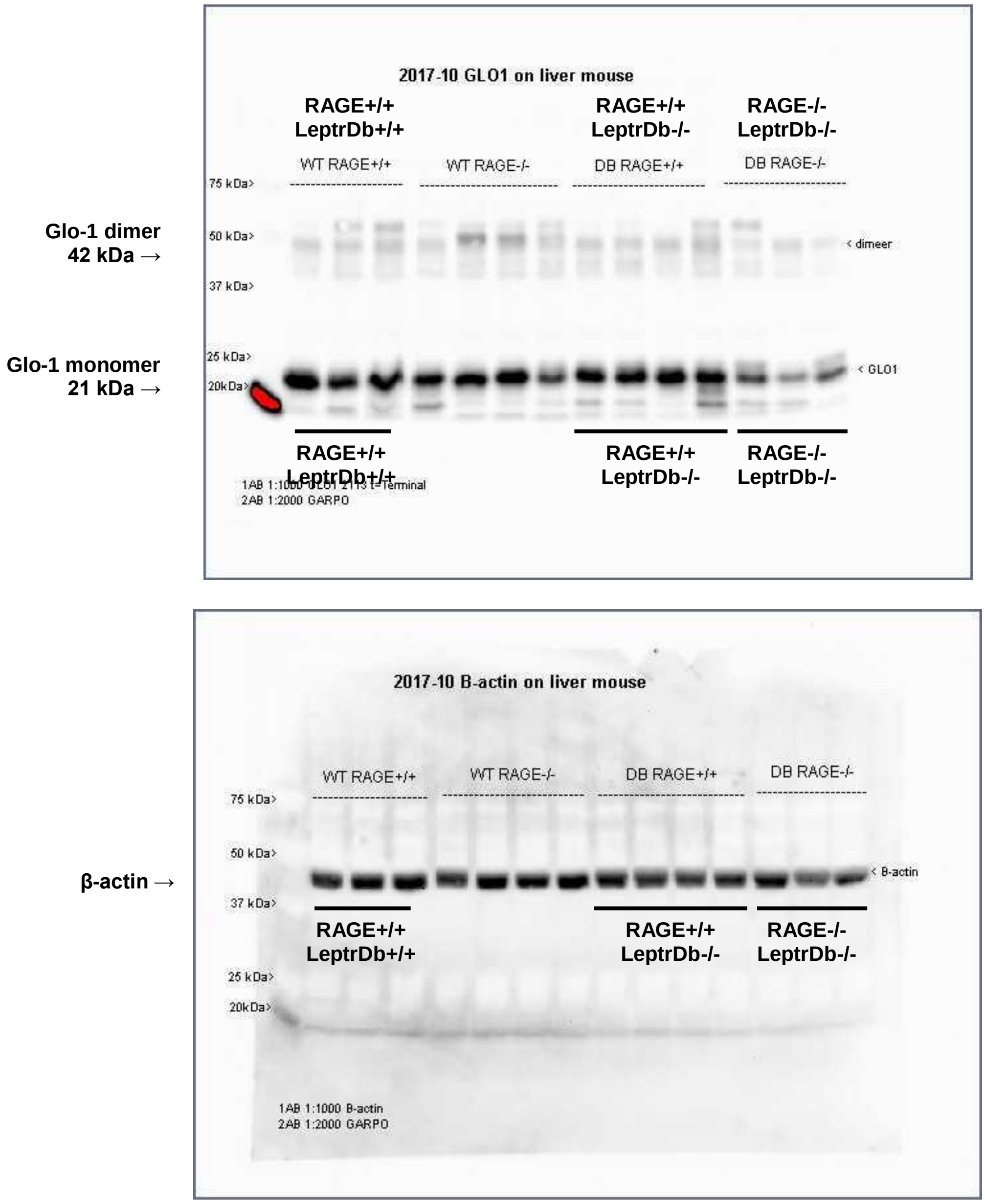
2017-10 GLO1 on liver mouse
RAGE+/+ LeptrDb+/+
RAGE+/+ LeptrDb-/-
RAGE-/- LeptrDb-/-
WT RAGE+/+
WT RAGE-/-
DB RAGE+/+
DB RAGE-/-
75 kDa
50 kDa
37 kDa
25 kDa
20kDa
< dimeer
< GLO1
Glo-1 dimer 42 kDa →
Glo-1 monomer 21 kDa →
RAGE+/+ LeptrDb+/+
RAGE+/+ LeptrDb-/-
RAGE-/- LeptrDb-/-
2AB 1:2000 GARPO
2017-10 B-actin on liver mouse
WT RAGE+/+
WT RAGE-/-
DB RAGE+/+
DB RAGE-/-
75 kDa
50 kDa
37 kDa
25 kDa
20kDa
< B-actin
β-actin →
RAGE+/+ LeptrDb+/+
RAGE+/+ LeptrDb-/-
RAGE-/- LeptrDb-/-
1AB 1:1000 B-actin
2AB 1:2000 GARPO

Figure 5.

B

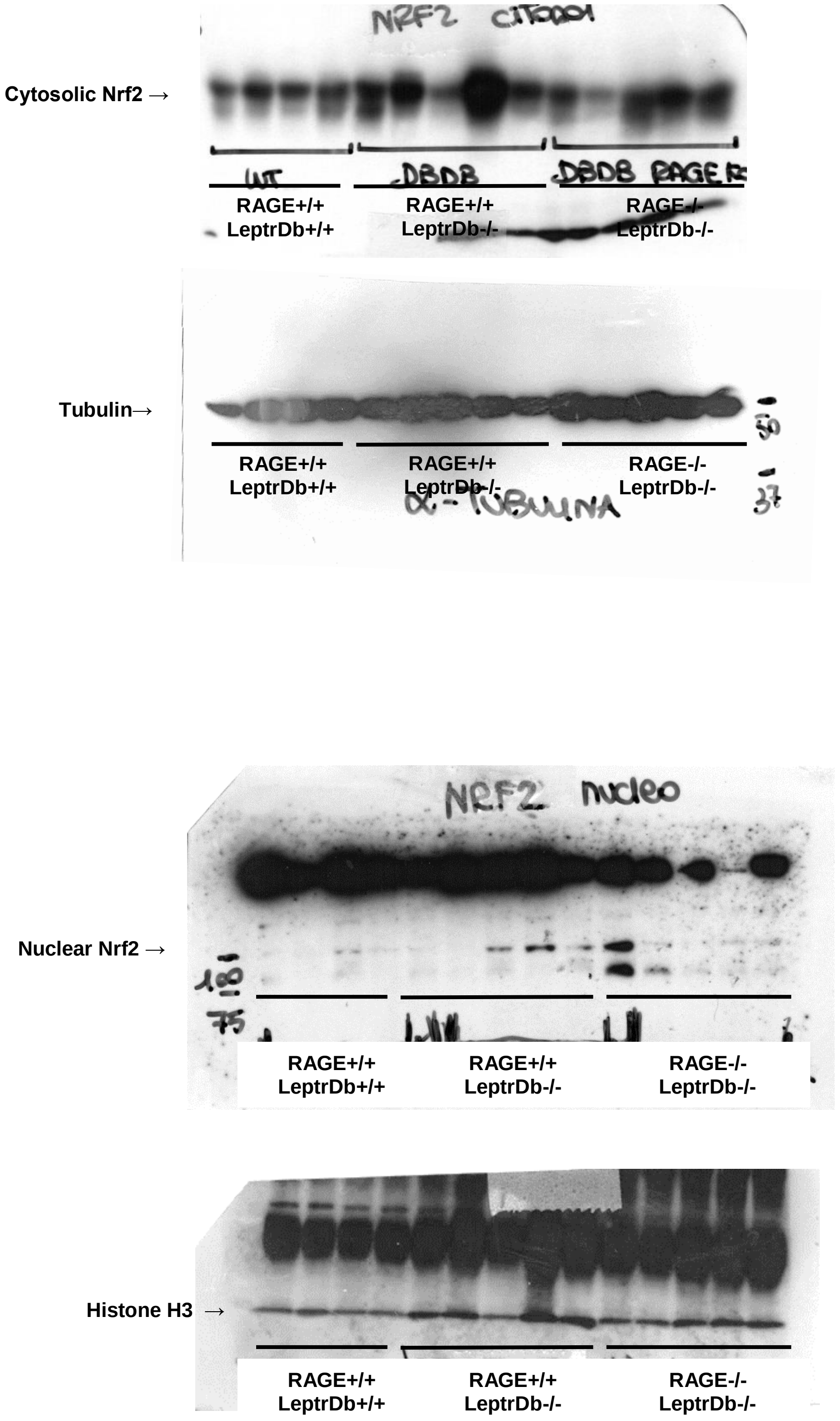

Cytosolic Nrf2 →
RAGE+/+ LeptrDb+/+
RAGE+/+ LeptrDb-/-
RAGE-/- LeptrDb-/-
Tubulin→
RAGE+/+ LeptrDb+/+
RAGE+/+ LeptrDb-/-
RAGE-/- LeptrDb-/-
Nuclear Nrf2 →
RAGE+/+ LeptrDb+/+
RAGE+/+ LeptrDb-/-
RAGE-/- LeptrDb-/-
Histone H3 →
RAGE+/+ LeptrDb+/+
RAGE+/+ LeptrDb-/-
RAGE-/- LeptrDb-/-

C

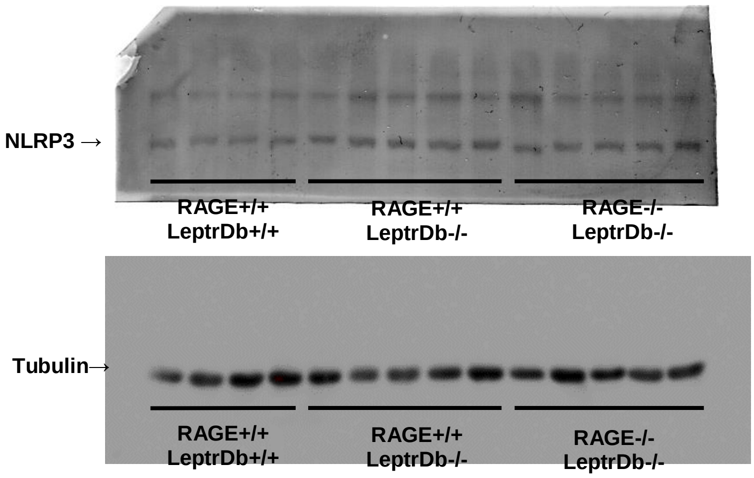
NLRP3 →
RAGE+/+
LeptrDb+/+
RAGE+/+
LeptrDb-/-
RAGE-/-
LeptrDb-/-
Tubulin→
RAGE+/+
LeptrDb+/+
RAGE+/+
LeptrDb-/-
RAGE-/-
LeptrDb-/-

D

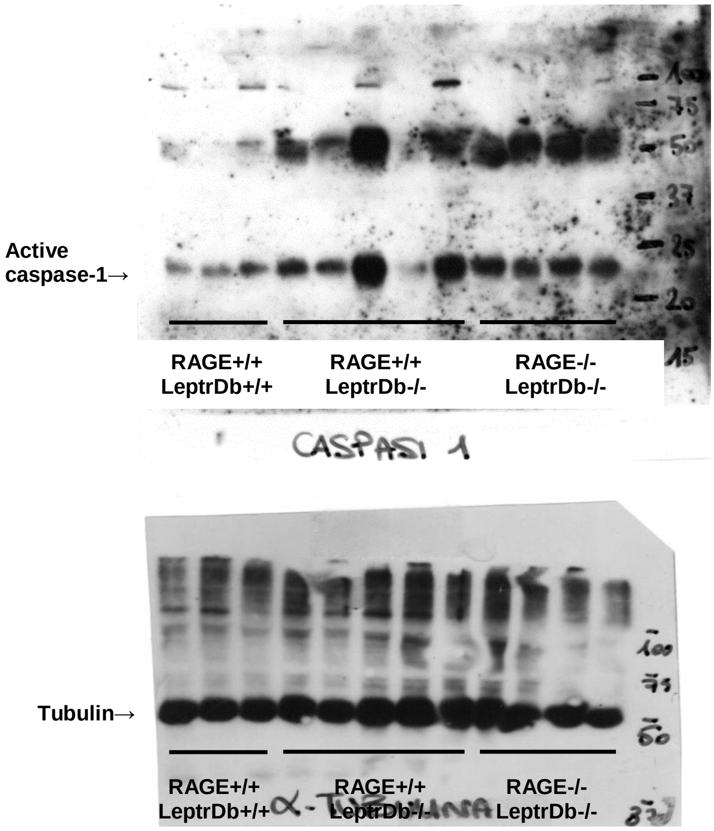
Active
caspase-1→
RAGE+/+
LeptrDb+/+
RAGE+/+
LeptrDb-/-
RAGE-/-
LeptrDb-/-
CASPASI 1
Tubulin→
RAGE+/+
LeptrDb+/+
RAGE+/+
LeptrDb-/-
RAGE-/-
LeptrDb-/-

E

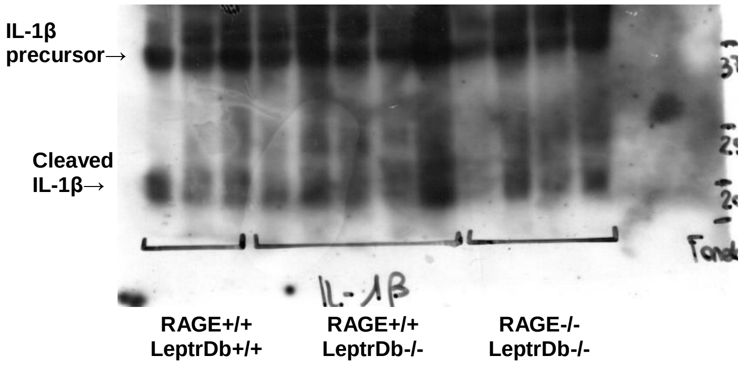
IL-1β
precursor→
Cleaved
IL-1β→
IL-1β
RAGE+/+
LeptrDb+/+
RAGE+/+
LeptrDb-/-
RAGE-/-
LeptrDb-/-

F

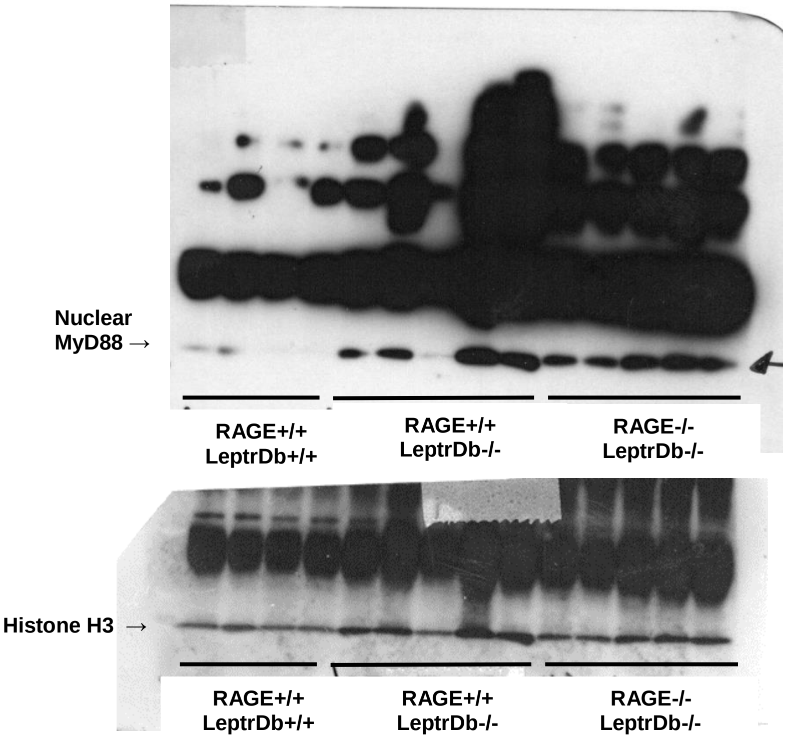
Nuclear
MyD88 →
RAGE+/+
LeptrDb+/+
RAGE+/+
LeptrDb-/-
RAGE-/-
LeptrDb-/-
Histone H3 →
RAGE+/+
LeptrDb+/+
RAGE+/+
LeptrDb-/-
RAGE-/-
LeptrDb-/-
